# Supplementary material for: Identification of 4,5,6,7-Tetrabromo-1H-benzotriazole (TBB) as a Small Molecule MESH1 Inhibitor that Suppresses Ferroptosis
Source: bioRxiv. 2026 Feb 20:2026.02.19.706832. Preprint. [Version 1] doi: 10.64898/2026.02.19.706832 (PMC12934728; doi:10.64898/2026.02.19.706832)
Supplement: Supplement 1 [file media-1.pdf]

Identification of 4,5,6,7-Tetrabromo-1*H*-benzotriazole (TBB) as a Small Molecule MESH1 Inhibitor that Suppresses Ferroptosis

Alexander A. Mestre, Yunju Oh, Jianli Wu, Denise Dunn, Yasaman Setayeshpour, Ssu-Yu Chen, Chao-Chieh Lin, C. Skyler Cochrane, Pyeonghwa Jeong, Gibeom Nam, Chloe Markey, Daniel Reker, Scott R. Floyd, Jiyong Hong*, Pei Zhou*, Jen-Tsan Chi*

Supplemental Material

Table S1. X-ray data collection and refinement statistics of the MESH1-TBB Complex

	**MESH1/ TBB (9ZZ9)**
**Data collection**	
Space group	C 2 2 21
Cell dimensions	
a, *b*, *c* (Å)	72.4 78.37 62.7
α, β, γ (°)	90.00, 90.00, 90.00
Resolution (Å)	40.56 - 2.33 (2.413 - 2.33)
R_{merge}	0.2105 (1.071)
R_{pim}	0.06048 (0.2961)
$CC_{1/2}$	0.998 (0.891)
$I / \sigma I$	13.77 (3.10)
Completeness (%)	99.92 (100.00)
Redundancy	13.2 (13.9)
**Refinement**	
Resolution (Å)	2.33
No. reflections	7906 (783)
R_{work} / R_{free}	0.2081/ 0.2277
No. atoms	1455
Protein	1410
Ligand/ion	14
Water	31
B-factors	46.40
Protein	46.38
Ligand/ion	56.56
Water	42.83
R.m.s. deviations	
Bond lengths (Å)	0.002
Bond angles (°)	0.39
Ramachandran	
favored (%)	99.43
allowed (%)	0.57
outliers (%)	0.0

*Values in parentheses are for highest-resolution shell.

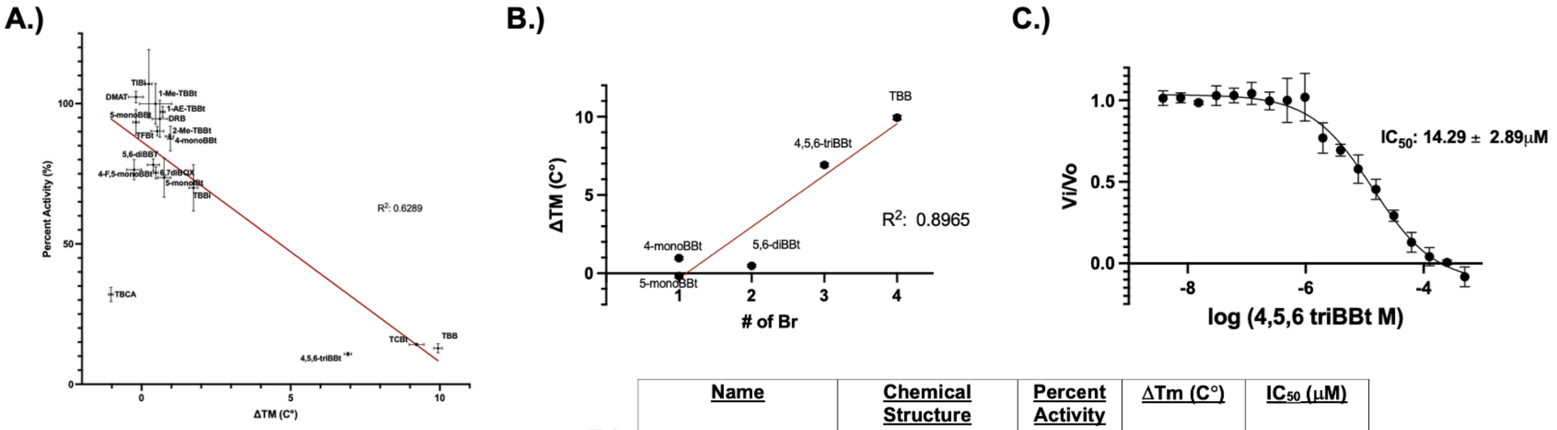

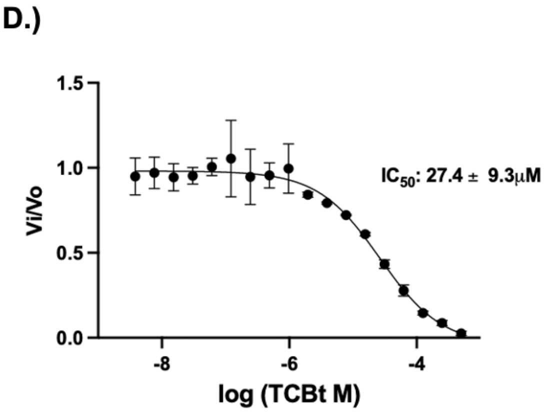

E.)

Name	Chemical Structure	Percent Activity at 100μM (%)	ΔTm (C°)	IC_{50} (μM)
TBB<br>4,5,6,7-tetrabromobenzotriazole	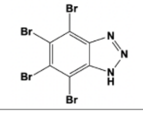	12.85 +/- 1.64	9.94 +/- 0.14	4.7 +/- 0.3
5-monoIBt<br>5-iodo-1H-1,2,3-benzotriazole	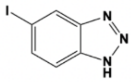	73.6 +/- 6.93	0.76 +/- 0.22	-
4-F,5-monoBBt<br>4-fluoro-5-bromo -1H-1,2,3-benzotriazole	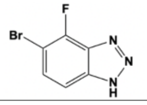	78.16 +/- 2.14	0.39 +/- 0.20	-
1-AE-TBBt<br>4,5,6,7-tetrabromo-1-(2-aminoethyl)benzotriazole	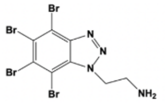	99.92 +/- 7.23	0.47 +/- 0.54	-
6,7diBQX<br>6,7-dibromoquinoxaline	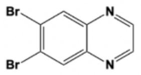	76.46 +/- 3.62	-0.25 +/- 0.25	-

Supplemental Figure 1. A. Correlation of enzyme inhibition (percent activity) and thermal shift (**Δ**Tm) at 100µM inhibitor. Data fitted with linear regression (n= 3 for both experiments, mean ± S.D). **B.** Correlation of # or Br and thermal shift (**Δ**Tm). Data fitted with linear regression (n= 3, mean ± S.D). **C.** IC50 curve of 4,5,6-triBBt fitted with four-parameter fitting (n= 3 independent experiments, mean ± S.D.) **D.** IC50 curve of TCBt fitted with four-parameter fitting (n= 3 independent experiments, mean ± S.D.). **E.** Table of other TBB analogs tested. In heat map red color = stronger inhibition and larger thermal shift and white = weaker inhibition and smaller thermal shift.

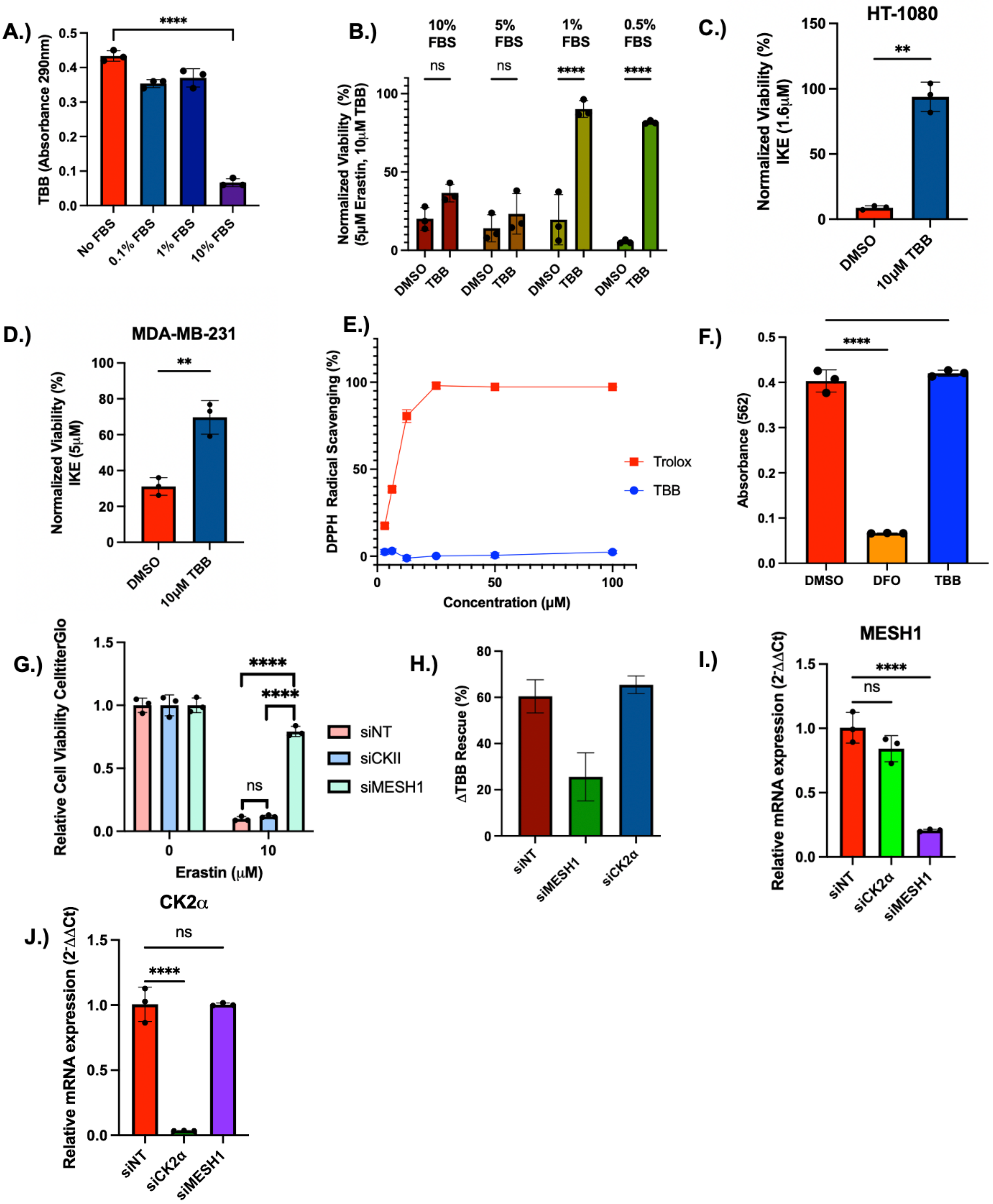

Supplemental Figure 2. A. Quantification of unbound TBB after incubation with various FBS concentrations. n=3 technical replicates, error bars = SD. One-way ANOVA with Dunnett's post-

hoc test vs. DMSO **B.** RCC4 cells were treated 5 µM erastin and DMSO or 10µM TBB at the indicated fetal bovine serum (FBS) concentrations and cell viability was determined with CellTiterGlo and normalized to control. n=3, error bars = SD. Two-way ANOVA with Sidák multiple comparisons (DMSO vs. TBB) **C-D.** CellTiter Glo assessment of cellular viability of HT-1080 and MDA-MB-231 treated with the combination of IKE and 10 µM TBB. n=3, error bars = SD. Unpaired two-tailed T-test (DMSO vs. TBB) **E.** DPPH radical scavenging activity of TBB and Trolox across the indicated concentrations. n=4 technical replicates error bars =SD. **F.** DMSO, DFO(100 µM) and TBB (100 µM) were incubated with Fe^{2+}, prior to the addition of ferrozine. A562nm was measured to quantify free Fe^{2+}.n=3, error bars = SD. One-way ANOVA with Dunnett's post-hoc test vs. DMSO **G.** CellTiter Glo measuring cell viability of HT-1080 cells treated with siNT, siCK2, and siMESH1 with or without the treatment of 10µM erastin.**H**. Quantification of TBB mediated rescue. **I, J.** RCC4 cells were transfected with siNT, siCK2α, siMESH1 for 48hours and mRNA levels were quantified by RT-PCR using GAPDH for normalization. Relative expression was calculated by 2^{-} ΔΔCt method. n= 3 biological replicates, error bars= SD. One-way ANOVA with Dunnett's post-hoc test vs. siNT

* = p-value <0.05, ** = p-value < 0.01, *** = p value <0.001, **** p-value < 0.0001

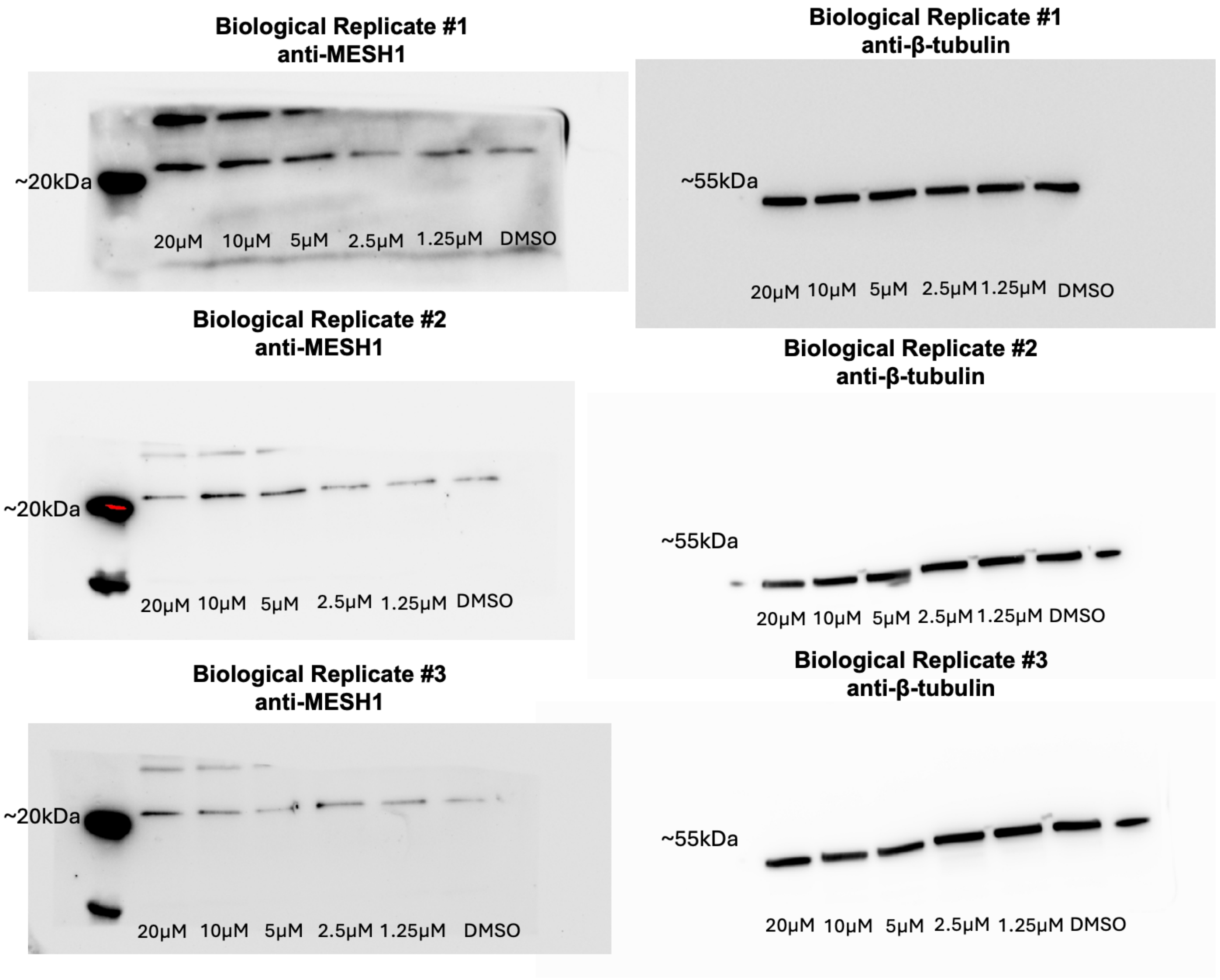

Supplemental Figure 3. Unprocessed Western Blots for Figure 4H. The MESH1 blot was captured at a longer exposure than the β-tubulin blot and the membrane outline of β-tubulin blots were not visible due to the short exposure.
